# Eosinophilic Esophagitis—Catching Up with the Hype Train: A Systematic Overview and Review of the Literature of the Emerging Disease

**DOI:** 10.3390/biomedicines13092230

**Published:** 2025-09-10

**Authors:** Jawad Hindy, Amir Mari, Tova Rainis, Gadeer A’li Taha

**Affiliations:** 1 Department of Gastroenterology and Hepatology, Bnai-Zion Medical Center, Haifa 3339419, Israel; tova.rainis@b-zion.org.il; 2 The Proteomic Unit, Bnai Zion Medical Center, Haifa 3339419, Israel; 3 Cancer Research Center, The Rappaport Faculty of Medicine, Technion, Israel Institute of Technology, Haifa 3525433, Israel; 4 Faculty of Medicine, Bar Ilan University, Ramat Gan 5290002, Israel; amir.mari@hotmail.com; 5 Department of Gastroenterology, EMMS Nazareth Hospital, Nazareth Hospital, Nazareth 1613101, Israel; 6 The Rappaport Faculty of Medicine, Technion, Israel Institute of Technology, Haifa 3525433, Israel; 7 Leumit Health Services, Tel Aviv-Yafo 6473817, Israel; dr.gadeertaha@gmail.com

**Keywords:** eosinophils, esophagus, inflammation, mucosa

## Abstract

**Background**: Eosinophilic esophagitis (EoE) is a chronic, immune-mediated esophageal disorder characterized by Th2-driven inflammation. Clinically, it manifests as esophageal dysfunction, including dysphagia and food impaction, and is frequently associated with atopic comorbidities. **Methods**: Diagnosis is established via histologic confirmation of ≥15 eosinophils per high-power field (hpf) on esophageal biopsy. Clinical presentation varies, ranging from subtle dysphagia to severe complications necessitating urgent endoscopic intervention. **Results**: Disease progression is characterized by esophageal remodeling, encompassing fibrosis, angiogenesis, and muscular hypertrophy. Management strategies require individualized, long-term approaches aimed at symptom control and prevention of structural complications. **Discussion**: Advances in the last decade have refined diagnostic criteria, standardized endoscopic scoring systems, and introduced novel therapeutic agents, including biologics. This review synthesizes current evidence regarding epidemiology, clinical manifestations, diagnostics, and therapeutic strategies.

## 1. Introduction

Eosinophilic Esophagitis (EoE) is a chronic, immune-mediated inflammatory disease of the esophagus, characterized by T-helper Type 2 (Th2)–driven inflammation. It can present across the lifespan, from childhood to late adulthood, and is defined by esophageal dysfunction with mucosal eosinophilia in the absence of alternative causes [1,2,3]. The precise pathogenesis remains incompletely understood, but available data—largely from observational studies and animal models—suggest complex interactions between genetic predisposition and environmental triggers [4]. While food allergens and impaired epithelial barrier function are consistently implicated [5], many studies are limited by small sample sizes and lack of longitudinal validation.

Cytokines such as IL-4, IL-5, and IL-13 are central mediators of disease; however, therapeutic trials targeting eosinophils have yielded inconsistent clinical results [6]. Variability in study design, sample size, and endpoints highlights the heterogeneity of the field and suggests that additional immune pathways contribute to pathogenesis.

Epidemiologically, EoE has shown a sharp rise in incidence and prevalence over the past two decades [7,8,9,10,11,12,13,14]. In North America and Europe, population-based studies report prevalence estimates ranging from 7 to 32.5 per 100,000 [9,13]. Methodological differences—including diagnostic thresholds and biopsy protocols—make direct comparison challenging. While increased awareness and endoscopic utilization partly explain the trend, studies suggest a true rise in incidence beyond diagnostic intensity [13,14]. Data from Asia, the Middle East, and other developing regions are limited; early reports suggest lower prevalence, but underdiagnosis and limited access to specialized pathology may contribute [15,16,17].

Symptomatology differs across age groups (Table 1): adults most commonly present with chronic, progressive dysphagia, while pediatric patients more often exhibit feeding difficulties or abdominal complaints [13]. Cultural and dietary practices likely influence disease manifestations, including food impaction patterns [18,19,20]. Most data on symptom burden and psychosocial impact are from Western cohorts [16,17,18,19], leaving non-Western populations underexplored.

In pediatric populations, the clinical presentation of EoE differs substantially from that observed in adults. Infants and toddlers often exhibit non-specific symptoms such as reflux-like complaints, vomiting, nausea, abdominal pain, food refusal, and failure to thrive (FTT) [20]. Symptom patterns evolve with age: failure to thrive is most commonly reported in younger children (median age ≈ 2 years), vomiting in school-aged children (≈8 years), abdominal pain in early adolescence (≈12 years), and dysphagia or food impaction in older adolescents (≈13–17 years) [21]. While these age-stratified associations are consistently described, most supporting data come from single-center or retrospective cohorts, which may introduce referral bias and limit generalizability. Additionally, diagnostic thresholds (e.g., eosinophil counts per high-power field) and symptom-reporting methodologies vary across studies, contributing to heterogeneity in reported prevalence of specific symptoms.

Complications of EoE arise primarily from defective remodeling of the inflamed esophagus, including fibrosis, angiogenesis, and smooth muscle hypertrophy [22]. Basal layer hyperplasia and progressive epithelial dysfunction contribute to luminal narrowing, stricture formation, and food impaction. Although these pathological processes are well-documented histologically, most evidence derives from cross-sectional biopsy analyses rather than longitudinal pediatric cohorts, leaving uncertainties regarding the pace and predictors of remodeling over time. Furthermore, the majority of natural history data originate from Western populations, whereas long-term outcomes in Asian, Middle Eastern, and resource-limited settings remain underexplored. Preliminary reports suggest regional variation in disease behavior, potentially influenced by diet, genetic background, and access to endoscopy, but the limited number and quality of these studies preclude definitive conclusions.

Taken together, while pediatric EoE is well-recognized to follow an age-dependent symptom trajectory and carries risk for long-term fibrostenotic complications, the evidence base remains methodologically heterogeneous and geographically skewed toward Western cohorts. Robust multicenter, longitudinal studies across diverse regions are needed to clarify risk factors for progression and to inform global management strategies.

## 2. Endoscopic Findings

**Figure 1:**
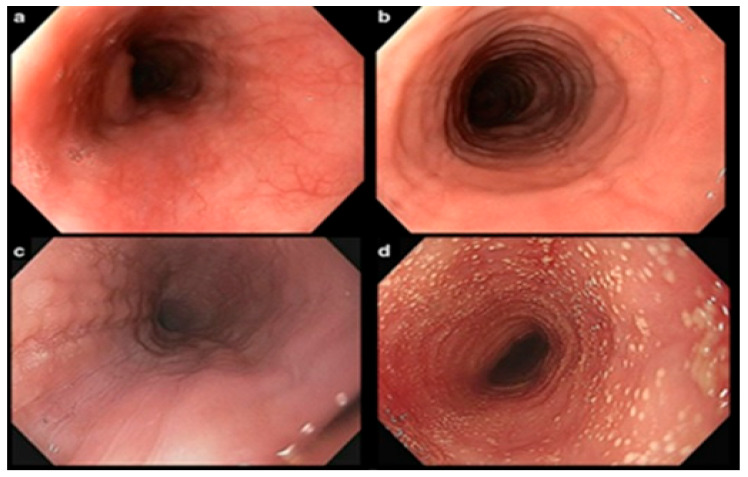
Endoscopic findings in patients with EoE: (**a**) Partial and soft mucosal edema. (**b**) Concentric rings and strictures of moderate severity. (**c**) Severe strictures with scattered whitish exudates localized within or adjacent to the narrowed areas and (**d**) Diffuse whitish exudates broadly covering most of the esophageal surface.

Although no single pathognomonic endoscopic finding defines EoE, esophagogastroduodenoscopy (EGD) remains the first diagnostic step in patients presenting with dysphagia for solids [23]. Commonly reported features include longitudinal furrows, white exudates, narrowings and strictures, trachealization, rings, edema, and “crepe paper” mucosa [24] (see Figure 1). These findings may appear in various combinations within the same patient. Acute inflammatory activity is generally reflected by white exudates, edema, and furrows, whereas fibrostenotic remodeling due to chronic inflammation is more often associated with rings, trachealization, and fixed stenosis.

To standardize reporting, the Eosinophilic Esophagitis Endoscopic Reference Score (EREFS) was developed, incorporating five major endoscopic features (edema, rings, exudates, furrows, and strictures) [25]. While EREFS has improved consistency in describing endoscopic abnormalities, its performance is not without limitations. Studies show inter-observer variability—with higher reproducibility for fixed rings and strictures, but only moderate agreement for subtler changes such as edema or exudates [24,25]. In terms of diagnostic accuracy, EREFS correlates with histologic eosinophilia, yet up to 10–15% of patients with biopsy-proven EoE may demonstrate a normal-appearing esophagus, reducing sensitivity. Conversely, certain EREFS features, such as rings or edema, are not exclusive to EoE and may occur in GERD or other esophageal disorders, affecting specificity [26].

Clinical correlation remains another challenge. Higher EREFS scores are generally associated with more severe disease and fibrostenotic complications, but correlations with patient-reported symptoms such as dysphagia are inconsistent, likely due to the multifactorial nature of esophageal dysfunction [27]. Furthermore, most validation studies of EREFS have been conducted in Western populations; data from Asia, the Middle East, and resource-limited settings are sparse, and differences in training, endoscopic equipment, and dietary exposures may influence scoring reliability.

Our group recently proposed expanding the Eosinophilic Esophagitis Endoscopic Reference Score (EREFS) to include esophagitis as a sixth feature, based on evidence that it may represent an additional manifestation of EoE not captured by the original framework [26]. This modification could enhance diagnostic sensitivity, but its validity requires confirmation in larger, multicenter cohorts before widespread adoption.

## 3. Histopathologic Features

**Figure 2:**
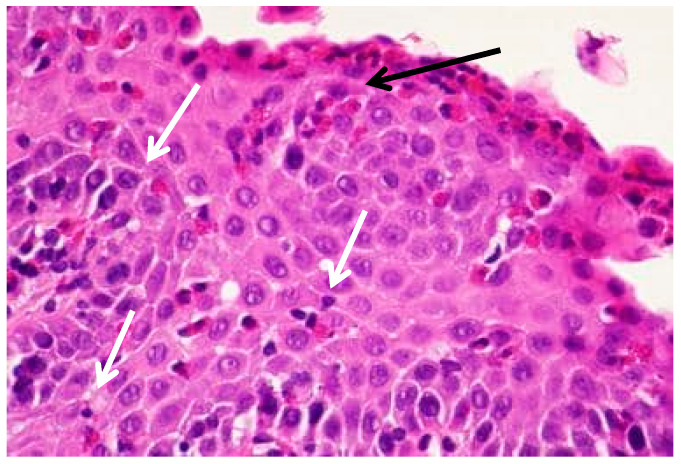
Histology from the esophagus of a patient with EoE stained with H&E. Figure 2 demonstrates the tissue before treatment with PPI, showing infiltration of numerous eosinophils in the mucosa (white arrows) with the formation of eosinophilis micro-abscess (black arrow) at 40x magnification.

**Figure 3:**
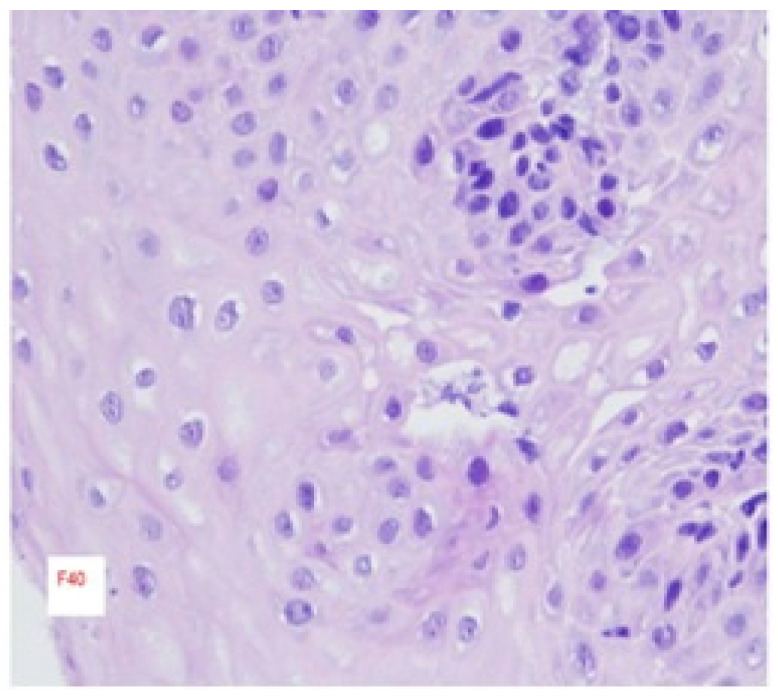
Histology from the esophagus of a patient with EoE stained with H&E. Figure 3 demonstrates the same tissue after 12 weeks of PPI treatment, where there is no presence of eosinophils or any other histologic characteristic of an active disease.

The histological hallmark of EoE is the infiltration of the esophageal epithelium by eosinophils, which often aggregate beneath the surface epithelium and may form micro-abscesses (Figure 2 and Figure 3) [2]. However, EoE involves more than eosinophils; it encompasses a complex inflammatory milieu including diverse lymphocyte subsets and mast cells, as well as contributions from the inflamed epithelial lining itself. Additional histopathological features associated with the inflammatory cascade include expansion of intercellular spaces, basal layer hyperplasia, and papillary elongation, which are typically seen in acute disease [26]. Eosinophilic infiltration often occurs at a later stage and contributes to tissue repair, ultimately leading to fibrosis in chronic disease [27].

To standardize and quantify these microscopic alterations, the EoE Histology Scoring System (EoEHSS) was developed [28]. This tool allows semi-quantitative assessment of both the severity and extent of histological features, improving reproducibility, diagnostic accuracy, and monitoring of treatment response. Nevertheless, several limitations remain. Inter-observer variability can be significant, particularly for features such as basal layer hyperplasia or papillary elongation, which are subject to subjective interpretation [29]. Differences in biopsy protocols—such as number of samples, location along the esophagus, and orientation of specimens—further contribute to variability across studies [30].

Evidence supporting EoEHSS primarily comes from retrospective or single-center cohorts in Western populations, limiting generalizability. Correlation between histologic scores and clinical symptoms is imperfect: some patients with high histologic severity may report minimal dysphagia, whereas others with low histologic scores experience significant symptoms [31]. These findings underscore the need to integrate histologic assessment with endoscopic and clinical data for comprehensive disease evaluation. Moreover, limited data are available from Asian, Middle Eastern, and developing-country cohorts, leaving open questions regarding regional differences in histologic presentation and progression.

## 4. Differential Diagnosis of Esophageal Eosinophilia and EoE Mimickers

Because esophageal eosinophilia (≥15 eosinophils/high-power field) is not pathognomonic, EoE remains a clinicopathologic diagnosis requiring both symptoms of esophageal dysfunction and exclusion of alternative causes of eosinophilia [28,29]. Multiple conditions can mimic EoE, each with distinct clinical, endoscopic, and histologic features; however, the supporting evidence varies in quality, and most data derive from single-center or retrospective Western cohorts.

Gastroesophageal Reflux Disease (GERD): GERD can produce low-grade, predominantly distal eosinophilia. Features favoring GERD include typical heartburn/regurgitation, Los Angeles–grade erosive esophagitis, and response to anti-reflux therapy [28,29]. However, overlap exists: persistent symptoms and ≥15 eos/hpf despite optimized reflux control favor EoE. Diagnostic accuracy is limited by heterogeneity in biopsy protocols and the lack of standardized thresholds across studies.

Infectious esophagitis (Candida, HSV, and CMV): Immunosuppressed patients with odynophagia, plaques, or ulcers may harbor infectious etiologies. Histology demonstrates organisms (GMS/PAS for fungi; viral cytopathic changes). Dense tissue eosinophilia is uncommon unless a secondary allergic component is present. Evidence mainly comes from case series, limiting generalizability [30].

Drug-/pill-induced esophagitis: Acute mid-esophageal ulcers following ingestion of medications such as doxycycline, bisphosphonates, or NSAIDs may mimic EoE. Biopsies typically show mixed inflammation rather than dense eosinophilia [31]. The temporal relationship is critical, but most reports are observational or anecdotal.

Non-EoE Eosinophilic Gastrointestinal Disorders (EGIDs): Multi-segment involvement with peripheral eosinophilia or elevated serum IgE suggests systemic EGID rather than isolated EoE [32]. Evidence for esophageal involvement outside EoE is limited, often derived from small case series or pediatric cohorts.

Systemic eosinophilic disorders (e.g., HES, EGPA): Marked peripheral eosinophilia and multi-organ involvement guide diagnosis, often supported by laboratory testing (AEC, FIP1L1-PDGFRA) [32]. Most published data are descriptive and retrospective.

Inflammatory bowel disease (IBD): Rare esophageal involvement occurs with aphthous or serpiginous ulcers and concomitant intestinal disease [30]. Evidence is primarily case-based.

Connective tissue and autoimmune disorders: Scleroderma, pemphigoid/pemphigus, lichen planus, and GVHD may mimic EoE. Clinical features, serology, and histology help differentiate, but reports are mostly small series [31].

Structural/motility disorders: Dysphagia from achalasia or spastic disorders may overlap clinically. Manometry or EndoFLIP differentiates these from EoE; eosinophilia is usually absent or mild [31,33].

Caustic or radiation injury; postsurgical strictures: Exposure history and endoscopic features (ulceration, necrosis, short smooth strictures) distinguish these from EoE [31].

Malignancy: Progressive dysphagia with mass or ulceration requires targeted biopsy; dense eosinophilia is typically absent [31].

Lymphocytic esophagitis: Can mimic EoE endoscopically, but histology shows peripapillary intraepithelial lymphocytosis with few eosinophils [30,31].

### Practical Clues Favoring EoE

Evidence-based features supporting EoE over mimickers include:

- Clinical: Atopy, food impaction, seasonal symptom fluctuation, pediatric feeding dysfunction, or failure to thrive [28,29,34]. Evidence is mostly observational; prospective studies linking these features to diagnostic accuracy are limited.
- Endoscopic: Multifocal EREFS features—edema, concentric rings, exudates, furrows, strictures—especially in the proximal/mid esophagus [28]. Inter-observer variability and regional differences in endoscopic practice may affect reliability.
- Histologic: Peak eosinophil count ≥ 15 eos/hpf, microabscesses, surface layering, basal zone hyperplasia, and lamina propria fibrosis [1,28,29]. Histologic thresholds and biopsy protocols vary across centers, contributing to heterogeneity.
- Therapeutic response: Improvement with dietary elimination, topical steroids, or PPI is supportive but not specific; histologic confirmation on follow-up improves diagnostic certainty [28,29].

Overall, the differential diagnosis of esophageal eosinophilia requires careful integration of clinical, endoscopic, and histologic data, with recognition of heterogeneity in study design, diagnostic criteria, and patient populations.

## 5. Diagnostic Criteria

To increase the likelihood of an accurate diagnosis, international guidelines recommend taking at least six separate biopsy samples from the distal and mid-proximal esophagus to avoid overlooking the disease [1]. There are three main criteria for the diagnosis of eosinophilic esophagitis:

- Prolonged symptoms caused by a dysfunctional esophagus.
- Primary histologic criterion: Peak eosinophil density of ≥15 eosinophils per high-power field (≈60 eos/mm^2^**)** within the squamous epithelium of the esophagus.Supportive features: Basal cell hyperplasia (often >20–30% of epithelial thickness; graded semiquantitatively), eosinophil micro-abscesses, eosinophil surface layering, eosinophil degranulation, dilated intercellular spaces (spongiosis)**,** and lamina propria fibrosis when subepithelial tissue is present. 2
- Exclusion of other causes of eosinophilic tissue infiltration, primarily GERD (Gastroesophageal Reflux Disease), connective tissue diseases, and Crohn’s disease.

## 6. Motility Assessment Tests

### 6.1. High-Resolution Manometry

Long-standing transmural inflammation in EoE can alter esophageal motility and compliance [35]. High-resolution manometry (HRM) is the gold standard for assessing esophageal motility and lower esophageal sphincter function [36]. Bassett et al. [37] found that 23% of EoE patients had nonspecific motor disorders, while 77% had normal motility. HRM may thus be useful in evaluating symptomatic refractory cases, especially in patients in histological remission without strictures.

### 6.2. EndoFLIP

Endoluminal functional lumen imaging probe (EndoFLIP) is not a standard diagnostic tool for EoE but may help identify strictures and assess fibrostenotic burden. Nicodeme et al. [38] showed that a distensibility plateau <225 mm^2^ (<17 mm diameter at 70 mL) predicted food impaction and the need for dilation over 4–12 months. EndoFLIP thus provides functional insight into esophageal stiffness, though further studies are needed to define its clinical utility and standardized thresholds.

## 7. Management

EoE is a chronic, progressive disorder requiring a lifelong management strategy aimed at preventing long-term complications. The therapeutic goals are to control symptoms, suppress esophageal inflammation, and preserve esophageal structure and function [39]. Treatment strategies include dietary modification, pharmacological therapy (PPIs and corticosteroids), biologic agents, and endoscopic dilatation (Table 2). The optimal choice depends on disease phenotype (inflammatory versus fibrostenotic), patient age and comorbidities, therapy accessibility and cost, and patient/clinician preference. Importantly, the evidence base is heterogeneous: most trials are Western, short-term, and use varying definitions of histological remission, making direct comparison across studies challenging [40].

### 7.1. Dietary Treatment

Dietary therapy remains the only approach that directly targets the causal trigger—food antigens driving mucosal inflammation. While capable of inducing clinical and histological remission, adherence and long-term sustainability remain key challenges.

- Elemental Diet: The landmark 1995 study by Kelly et al. [41]. demonstrated remission in 10 children using amino-acid–based formula. Subsequent series confirmed high efficacy (>90% remission), but real-world use is limited by poor palatability, weight loss, high cost, and psychosocial burden [42]. Most studies are pediatric and small in scale, limiting generalizability.
- Empiric Elimination Diets (2-4-6 food): Empiric elimination of common food allergens (milk, wheat, egg, soy, nuts, seafood) achieves histologic remission in ~50–70% of patients [43]. Outcomes vary by the number of foods eliminated and by region: dairy is the most frequent trigger in Western cohorts, whereas wheat and soy predominate in some Asian series, highlighting geographic heterogeneity.
- Allergy Test–Directed Diets: Diets guided by skin-prick or patch testing have lower and inconsistent efficacy (~30–45% remission), as predictive value of allergy testing for EoE triggers is limited [44]. Evidence is derived largely from small observational cohorts, with variable methodology.

Overall, dietary therapy is effective but limited by adherence, nutritional risks, and quality-of-life impact. Long-term data are sparse, and global practice patterns differ markedly.

### 7.2. Proton Pump Inhibitors (PPIs)

Although EoE is immunologically distinct from GERD, PPIs demonstrate efficacy via both acid suppression and anti-inflammatory effects (reducing eotaxin-3 expression) [43,45,46]. Remission rates vary (30–50%) with substantial heterogeneity across trials in design, inclusion criteria, and outcome definitions.

PPIs are widely used as first-line therapy in adults, typically initiated at high-dose twice daily for 8–12 weeks, with follow-up endoscopy to assess histological response. Tapering to the lowest effective maintenance dose is common in long-term management.

Safety concerns—nutrient deficiencies, infections, kidney disease, and bone health—stem primarily from observational data in non-EoE populations [47,48,49,50]. Causality remains debated, and absolute risks are low. Long-term EoE-specific safety studies are lacking.

### 7.3. Topical Corticosteroids

Corticosteroids (CS) possess anti-inflammatory properties, reduce esophageal constriction, and enhance the integrity of the esophageal barrier. Both systemic and topical steroids are effective in inducing histological remission in Eosinophilic Esophagitis (EoE) patients across all age groups [51]. However, systemic administration can cause various side effects, including hyperphagia, weight gain, adrenal suppression, growth retardation, osteopenia, osteoporosis, esophageal candidiasis, glucose intolerance, and cataract formation [51]. Topical corticosteroids, such as budesonide and fluticasone, are highly effective in inducing histological remission and improving symptoms, with response rates up to 90% in randomized controlled trials (RCTs) [52]. Formulations that maximize mucosal contact time, such as viscous suspensions or orally disintegrating tablets, further enhance efficacy [53].

- Budesonide Orally Disintegrating Tablet (BOT, Jorveza): In phase III RCTs, Jorveza 1 mg twice daily induced histological and clinical remission in approximately 85–90% of patients at 12 weeks [54]. Maintenance trials (48 weeks, *n* = 204) demonstrated sustained remission in about 75% on 1 mg daily, with relapse upon discontinuation [55].
- Safety: The most frequent adverse effect is esophageal/oral candidiasis (~10–15%), typically mild and treatable [54,55]. Long-term studies indicate minimal systemic absorption, with no significant adrenal suppression, osteoporosis, or metabolic side effects [56,57]. Nonetheless, guidelines recommend periodic monitoring [56].

The evidence supporting these treatments is robust, based on multiple RCTs; however, most studies have been short-term (<1 year) and adult-focused. Pediatric data remain limited.

### 7.4. Biologic Therapy

Biologics represent the newest frontier in EoE management.

- Anti-IL-5 agents (mepolizumab, reslizumab): Early randomized controlled trials (RCTs) demonstrated reductions in esophageal eosinophilia but no consistent clinical benefit, limiting their current role; trial designs were often small, heterogeneous, and predominantly focused on pediatric populations [57,58].
- Dupilumab (anti–IL-4Rα): This agent blocks IL-4 and IL-13 signaling—central to Th2-mediated inflammation. In phase III RCTs in adolescents and adults, weekly subcutaneous Dupilumab significantly improved dysphagia scores and reduced esophageal eosinophilia versus placebo [59]. Long-term extension studies confirm sustained efficacy and no new safety signals. The most commonly reported adverse events include injection-site reactions, mild conjunctivitis, and transient eosinophilia [59].
- Regulatory approval and guidelines: Dupilumab is now approved in the US, Europe, and other regions as the first biologic for EoE [60]. Some guidelines continue to recommend topical steroids as first-line due to cost considerations, while others endorse dupilumab as a first-line option for patients with severe disease or overlapping atopic comorbidities (e.g., asthma, dermatitis, chronic rhinosinusitis with nasal polyps) [60].

Overall, research on biologic therapies in EoE remains strong yet limited to a few agents and largely Western cohorts. Access and cost are significant global barriers.

### 7.5. Endoscopic Dilatation

Endoscopic balloon dilatation addresses fibrostenotic complications such as strictures and rings. Symptomatic improvement occurs in approximately 75% of patients, often immediately, with durability up to 12 months [61]. However, dilatation does not reduce underlying inflammation and should be combined with medical therapy.

Complication rates are low (<1% perforation or bleeding) [62], though the risk is not negligible. Histologic remission appears to reduce the need for repeat dilatation, underscoring the importance of combining anti-inflammatory and mechanical strategies.

## 8. Natural History

In EoE, the course is relatively benign, often with mild dysphagia as the predominant symptom in most patients. About 30% to 50% of affected children report dysphagia symptoms beginning in adolescence, leading them to adapt their eating habits, such as avoiding certain foods (e.g., meat, crusty bread), increasing fluid intake during meals, and prolonging mastication before swallowing. As the disease progresses slowly, strictures in the esophagus typically appear about 10 years after symptom onset, affecting approximately half of patients [63,64].

Longitudinal studies have demonstrated that even short-term pharmacological or dietary interventions can provide several months of symptom relief [64,65]. Additionally, esophageal dilation has been shown to successfully alleviate dysphagia symptoms, even without pharmacological treatment [65]. Spontaneous remission does occur but is uncommon. Importantly, the duration of untreated active inflammation appears to be the best predictor for the development of esophageal strictures [63,64].

Therefore, early and optimal treatment may slow disease progression; however, supporting data are limited, and few factors reliably predict the rate of progression to the fibrotic stage or the likelihood of esophageal stenosis [65].

## 9. Conclusions

Eosinophilic Esophagitis (EoE) is a chronic, progressive disorder that has emerged as a significant clinical entity due to its increasing prevalence and recognition. Timely identification of affected individuals through comprehensive evaluation—including esophagogastroduodenoscopy with systematic, multi-segment biopsies—is essential for accurate diagnosis. Early and appropriate therapeutic interventions not only reduce esophageal eosinophilic inflammation but also play a critical role in preventing long-term sequelae, including fibrostenotic remodeling, strictures, and food impaction. These observations underscore the importance of heightened clinical vigilance, prompt diagnosis, and individualized management strategies to mitigate disease progression and improve patient outcomes. Despite advances in diagnostic and therapeutic approaches, further research is required to optimize long-term management, clarify the role of emerging biologic therapies, and identify reliable predictors of disease progression.

## Figures and Tables

**Table 1:** Clinical manifestations in adults versus children [20,21].

Adult	Children
Dysphagia	GERD-like symptoms
Food impaction	Failure to thrive
Chest pain	Food refusal
GERD	Vomiting
Regurgitation	Abdominal pain
Abdominal pain	Over mastication of food

**Table 2:** Summary of the main therapeutic approaches for EoE, including dietary, pharmacologic, biologic, and endoscopic interventions. For each treatment, key advantages and limitations are listed based on efficacy, safety, and practicality. This table is intended to provide a concise overview for clinicians and researchers regarding the selection and expected outcomes of available therapies.

Treatment	Advantages	Disadvantages
**Dietary Treatment**	Targets underlying cause (food antigens); can achieve complete clinical and histologic remission; non-pharmacologic	Difficult adherence, social/psychological burden, weight loss with elemental diets; multiple endoscopies required for stepwise elimination
**Proton Pump Inhibitors (PPIs)**	Widely available; effective in 30–50% of patients; addresses acid reflux and decreases eotaxin-3; safe in most patients	Not effective in all; potential long-term risks (C. difficile, infections, nutrient deficiencies, kidney disease, osteoporosis); requires follow-up biopsies
**Topical Corticosteroids** (Budesonide gel/tablets, Fluticasone)	High histologic remission (~70–90%); minimal systemic absorption; multiple formulations available; effective maintenance therapy	Local candidiasis (~10–15%), mild dysphonia, throat irritation, xerostomia; requires proper administration; long-term safety monitoring recommended
**Biologic Therapy** (Dupilumab, anti–IL-5 agents)	Highly effective for moderate-to-severe/refractory EoE; improves atopic comorbidities; favorable safety profile; subcutaneous dosing	Expensive; limited availability; injection-site reactions, URT infections, conjunctivitis; anti–IL-5 agents reduce Eosinophils but may not induce clinical remission
**Endoscopic Dilatation**	Rapid relief of strictures; effective in ~75% of patients; low complication rate (<1%)	Does not treat underlying inflammation; invasive; risk of perforation or bleeding; often requires repeat procedures

## Data Availability

Not applicable.
